# Imperfect pseudo-merohedral twinning in crystals of fungal fatty acid synthase

**DOI:** 10.1107/S0907444909000778

**Published:** 2009-01-20

**Authors:** Simon Jenni, Nenad Ban

**Affiliations:** a Institute of Molecular Biology and Biophysics, ETH Zurich, 8093 Zurich, Switzerland

**Keywords:** imperfect pseudo-merohedral twinning, fungal fatty acid synthase

## Abstract

The recent high-resolution structures of fungal fatty acid synthase (FAS) have provided new insights into the principles of fatty acid biosynthesis by large multifunctional enzymes. The crystallographic phase problem for the 2.6 MDa fungal FAS was initially solved to 5 Å resolution using two crystal forms from *Thermomyces lanuginosus*. Monoclinic crystals in space group *P*2_1_ were obtained from orthorhombic crystals in space group *P*2_1_2_1_2_1_ by dehydration. Here, it is shown how this space-group transition induced imperfect pseudo-merohedral twinning in the monoclinic crystal, giving rise to a Moiré pattern-like interference of the two twin-related reciprocal lattices. The strategy for processing the twinned diffraction images and obtaining a quantitative analysis is presented. The twinning is also related to the packing of the molecules in the two crystal forms, which was derived from self-rotation function analysis and molecular-replacement solutions using a low-resolution electron microscopy map as a search model.

## Abstract 2

A case of imperfect pseudo-merohedral twinning in monoclinic crystals of fungal fatty acid synthase is discussed. A space-group transition during crystal dehydration resulted in a Moiré pattern-like interference of the twinned diffraction patterns.

## 1. Introduction

Eukaryotic fatty acid synthases (FASs) are large multifunctional enzymes that catalyze all the reaction steps in the essential biosynthesis of fatty acids. Recent X-ray crystallo­graphic studies on the mammalian and fungal enzymes have established a structural basis for understanding the architectural organization, molecular function and evolution of these megasynthases. Fungal FAS is an α_6_β_6_ heterododeca­meric complex with a molecular weight of 2.6 MDa that forms a 27 nm barrel-shaped particle in which fatty acid production is compartmentalized in two large reaction chambers (Jenni *et al.*, 2006 ▶, 2007 ▶; Leibundgut *et al.*, 2007 ▶; Lomakin *et al.*, 2007 ▶; Johansson *et al.*, 2008 ▶), whereas mammalian FAS is an X-­shaped 540 kDa α_2_ homodimer with two semicircular reaction chambers on both sides of the molecule (Maier *et al.*, 2006 ▶, 2008 ▶).

**Figure 1:**
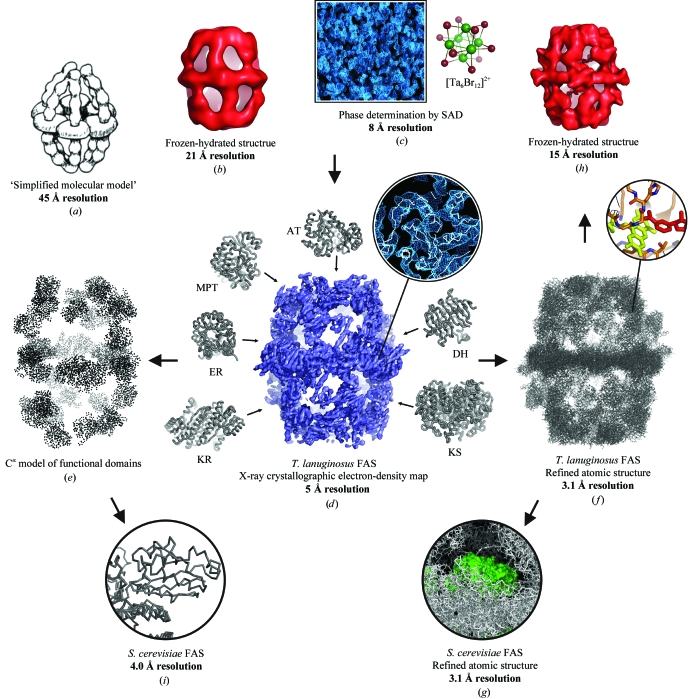
Structural studies of fungal fatty acid synthase (FAS). The figure summarizes the flow of phase information between the different X-ray crystallographic and electron-microscopic (EM) systems for structural determination of fungal FAS at progressively higher resolution. (*a*) Low-resolution model of the multi-enzyme based on a three-dimensional reconstruction from EM images of negatively stained particles (Hackenjos & Schramm, 1987 ▶). (*b*) Three-dimensional single-particle reconstruction at 21 Å resolution from frozen-hydrated particles (Kolodziej *et al.*, 1997 ▶). This EM map was used as a search model to derive molecular-replacement solutions for several crystal forms of fungal FAS (Jenni *et al.*, 2006 ▶; Xiong, 2008 ▶). (*c*) 8 Å resolution electron-density map of *T. lanuginosus* FAS after crystal derivatization with Ta_6_Br_12_ heavy-atom clusters and SAD phasing in space group *P*2_1_2_1_2_1_ (Jenni *et al.*, 2006 ▶). (*d*) Electron-density map in space group *P*2_1_ of *T. lanuginosus* FAS phased at 5 Å resolution by cross-crystal and noncrystallographic symmetry (NCS) density averaging starting with phases from (*c*). At this resolution, molecular features were recognized and the map was interpreted by rigid-body fitting of structures from homologous individual enzymes (Jenni *et al.*, 2006 ▶). (*e*) C^α^ model obtained from the fitted functional domains. (*f*) Refined atomic structure of FAS from *T. lanuginosus* (Jenni *et al.*, 2007 ▶). This model was employed to phase the yeast FAS crystal structure by molecular replacement (*g*) (Leibundgut *et al.*, 2007 ▶) and to initially determine the projection angles of class averages for the 15 Å resolution single-particle reconstruction of the enzyme (*h*) (Jenni *et al.*, 2007 ▶). (*i*) Phases calculated from a molecular-replacement solution obtained from the C^α^ model were used to solve the structure of yeast FAS at 4 Å resolution (Lomakin *et al.*, 2007 ▶).

Although the crystallization of yeast FAS was reported almost 40 years ago (Oesterhelt *et al.*, 1969 ▶), it was not until recently that structural information was extracted from crystals of this large assembly. This was because solution of the structures of crystals with large asymmetric units presents significant challenges. Such difficulties can now be overcome by the advancement of methods in macromolecular crystallo­graphy and the ability to collect data from weakly diffracting crystals using X-ray beams from synchrotron-radiation sources (Mueller *et al.*, 2007 ▶). An important step towards the atomic structure of fungal FAS was the solution of the crystallo­graphic phase problem, which yielded interpretable electron-density maps at 5 Å resolution from *Thermomyces lanuginosus* FAS (Jenni *et al.*, 2006 ▶). This phase information subsequently allowed further X-ray crystallographic and electron-microscopic (EM) studies of *Saccharomyces cere­visiae* FAS (Fig. 1 ▶).

To reliably identify the folds of individual enzymatic FAS domains in crystallographic electron-density maps, it was necessary to reach a resolution of about 5 Å, at which α-­helices are clearly resolved and β-sheets are visible as flat surfaces. *T. lanuginosus* FAS crystals that diffracted to this resolution were only obtained after dehydration, which occurred during stabilization. The improvement of the diffraction properties upon dehydration unfortunately also resulted in a space-group transition from orthorhombic to monoclinic, leading to imperfect pseudo-merohedral twinning, which complicated structure determination. Here, we describe how we identified the twinning in the monoclinic crystal form and how we processed the diffraction images acquired from twinned crystals, which allowed us to calculate accurate intensity statistics for a quantitative description of the twinning. We also show how molecular-replacement solutions were obtained for the two crystal forms at very low resolution, which established the packing of the FAS molecules and rationalized the observed twinning.

## 2. Materials and methods

### 2.1. Crystallization and data collection

The purification, crystallization and structural determination of FAS from *T. lanuginosus* have been described pre­viously (Jenni *et al.*, 2006 ▶). In brief, purified enzyme at a concentration of about 5 mg ml^−1^ in crystallization buffer containing 0.05 *M* tris(hydroxymethyl)aminomethane (tris) pH 7.6, 0.012 *M* magnesium acetate, 0.75 *M* potassium chloride, 0.002 *M* ethylenediaminetetraacetic acid (EDTA) pH 8.0 and 0.04 *M* β-mercaptoethanol was subjected to crystallization by the vapor-diffusion technique at 295 K. Equal volumes of protein-containing solution were mixed with reservoir solution consisting of 0.1 *M* bis(2-hydroxyethyl)­imino-tris(hydroxymethyl)methane (bis-tris) pH 5.8–6.2, 0.6 *M* sodium chloride and 3.8–5.0%(*w*/*v*) polyethylene glycol (PEG) 6000 (Jenni *et al.*, 2006 ▶). Crystallization conditions were further optimized by adding 3%(*w*/*v*) fructose and related sugars to the reservoir solution, by premixing enzyme-containing solution and reservoir solution in a separate test tube before dispensing the sample into the crystallization plate and by adding β-mercaptoethanol to a concentration of 0.2 *M* to the reservoir before sealing the plates (Jenni *et al.*, 2007 ▶). *T. lanuginosus* FAS crystals grew in a primitive orthorhombic space group and were stabilized by a gradual increase in the lithium sulfate concentration to a final concentration of 1.4 *M*. Primitive monoclinic crystals were obtained by stabilization in 0.1 *M* bis-tris pH 6.0, 0.05 *M* potassium chloride, 0.6 *M* sodium chloride, 0.012 *M* magnesium acetate, 23%(*w*/*v*) PEG 6000, 11%(*v*/*v*) 1,3-propanediol. Diffraction patterns were recorded on beamline X06SA at the Swiss Light Source (SLS) at 100 K.

### 2.2. Data processing

The data used for structure determination (Jenni *et al.*, 2006 ▶) were integrated and scaled using *XDS* (Kabsch, 1993 ▶). For the twinning analysis, data were integrated and scaled using *DENZO*/*SCALEPACK* (Otwinowski & Minor, 1997 ▶). Self-rotation and translation functions were calculated using the program *REPLACE* (Tong & Rossmann, 1997 ▶). The program *UNTANGLE* was used to remove overlapping reflections for the twinning analysis (Buts *et al.*, 2004 ▶).

## 3. Results and discussion

### 3.1. Molecular-replacement solution in the *P*2_1_2_1_2_1_ and *P*2_1_ crystals

The bipyramidal *T. lanuginosus* FAS crystals crystallized using PEG 6000 as precipitant and reached typical final dimensions of 0.2 × 0.3 × 0.4 mm (Jenni *et al.*, 2006 ▶). To improve crystal quality for data collection, we applied various post-crystallization methods. Screening of different crystal-stabilization and cryoprotection protocols ultimately yielded two populations of crystals that differed in space group and diffraction properties. The primitive orthorhombic crystals belonged to space group *P*2_1_2_1_2_1_, with unit-cell parameters *a* = 248, *b* = 379, *c* = 420 Å, and diffracted to 8 Å resolution. Primitive monoclinic crystals belonging to space group *P*2_1_ with unit-cell parameters *a* = 217, *b* = 415, *c* = 222 Å, β = 112° were obtained after incubation with higher concentrations of PEG 6000, typically 23%(*w*/*v*), prior to freezing. These crystals displayed superior diffraction that extended beyond 5 Å resolution. Matthews coefficients (Matthews, 1968 ▶) suggested the presence of one complete FAS particle in the asymmetric unit of both crystal forms. The corresponding solvent contents were 67 and 65% for the orthorhombic and monoclinic space groups, respectively. A substantial improvement in crystal diffraction has been observed in numerous cases when crystals were dehydrated by raising the concentration of high-molecular-weight PEG in the mother liquor, but a space-group change upon dehydration is unusual (Heras & Martin, 2005 ▶).

**Figure 2:**
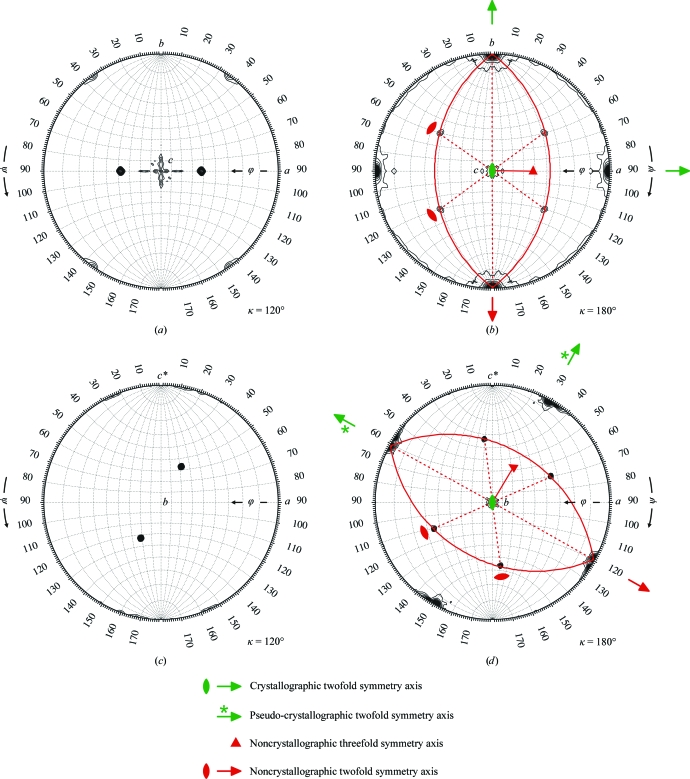
Self-rotation function analysis of native Patterson maps from *T. lanuginsosus* FAS crystals. (*a*, *b*) Orthorhombic *P*2_1_2_1_2_1_ crystal form. (*c*, *d*) Monoclinic *P*2_1_ crystal form. In the stereographic projections for κ = 180°, the directions of the threefold noncrystallographic symmetry (NCS) axis are indicated by red triangles. In the stereographic projections, the great circles, which represent the intersection of the plane perpendicular to the threefold NCS axis with the sphere, are shown in red.

Previous EM studies had established 32 symmetry for the fungal FAS particle (Hackenjos & Schramm, 1987 ▶; Kolodziej *et al.*, 1997 ▶). Self-rotation functions calculated from the native Patterson maps of the two crystal forms revealed the direction of the noncrystallographic symmetry (NCS) threefold and twofold axes of the FAS particles with respect to the crystallo­graphic symmetry axes (Fig. 2 ▶). Outstanding solutions were obtained for the threefold NCS axes. The twofold axes were initially difficult to identify, but could be located when we inspected the anticipated regions of the spherical polar space on the great circles perpendicular to the threefold axes (shown in red in Fig. 2 ▶) or when we calculated locked rotation functions (Tong & Rossmann, 1990 ▶) with 32 symmetry imposed. We made three important conclusions by comparing the self-rotation function solutions for the *P*2_1_ and *P*2_1_2_1_2_1_ crystals. Firstly, they are consistent and therefore both are likely to be correct. Because the monoclinic crystal form was obtained during stabilization from the orthorhombic form, we anticipated related symmetry in the Patterson functions of both crystals. Indeed, the *P*2_1_ crystal has strong pseudo-crystallo­graphic 222 symmetry that is reminiscent of true orthorhombic symmetry (Fig. 2 ▶ *d*). Moreover, the orientations of the FAS NCS axes with respect to the crystallographic and pseudo-crystallo­graphic twofold axes are virtually the same. This is best illustrated if a counterclockwise rotation of 60° is applied to Fig. 2 ▶(*b*), making it essentially congruent with Fig. 2 ▶(*d*). Secondly, there is definitely one complete FAS particle in the asymmetric unit of both crystal forms because none of the NCS axes align with the unique monoclinic twofold axis (Fig. 2 ▶ *d*) and the volume ratio of the *P*2_1_2_1_2_1_ and *P*2_1_ asymmetric units is 1.06. Thirdly, in the *P*2_1_2_1_2_1_ unit cell, which has four asymmetric units and contains four FAS particles, there are two pairs of asymmetric units in which the molecules assume the same orientation. This is because one twofold NCS axis is parallel to the crystallographic twofold screw *b* axis.

**Figure 3:**
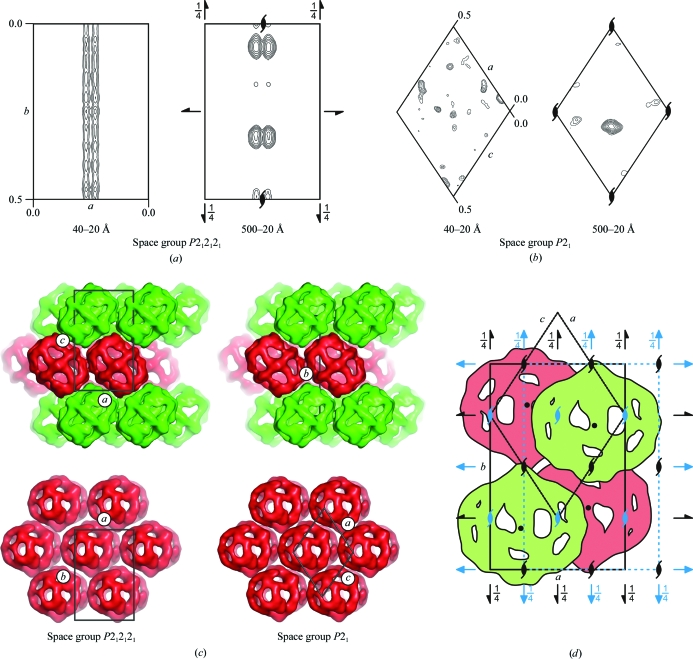
Molecular-replacement solutions for the two FAS crystal forms were obtained using a low-resolution EM reconstruction as search model. (*a*) Translation functions for the *P*2_1_2_1_2_1_ crystal calculated with two different resolution cutoffs. The section *z* = 0.25 of the asymmetric unit for the translation function (the Cheshire cell; Hirshfeld, 1968 ▶) is shown. (*b*) Translation functions for the *P*2_1_ crystal shown on the same scale. (*c*) Packing of the FAS molecules in the two crystal forms. (*d*) Pseudo-symmetry in the primitive orthorhombic FAS crystal form. The primitive orthorhombic unit cell and the twofold screw axes that define space group *P*2_1_2_1_2_1_ are shown in black. A *C*-centered orthorhombic unit cell with its additional symmetry in the *C*222_1_ space group is depicted in blue. The packing of the FAS molecules displays pseudo-*C*-centered orthorhombic symmetry. The space group is *P*2_1_2_1_2_1_ and not *C*222_1_ because the FAS molecule, with its center of mass indicated by a black dot, is offset by 5 Å along the *a* axis. The 5 Å offset along the *a* axis is also clearly visible as double peaks in the translation function shown in (*a*). A monoclinic unit cell is also shown with respect to the orthorhombic geometry and symmetry.

To complete the molecular-replacement solution, we oriented the 21 Å resolution FAS map (Kolodziej *et al.*, 1997 ▶) according to the self-rotation analysis and used it as a search model in the translation function (in fact, the self-rotation analysis allows two possible orientations related by a 60° rotation around the threefold NCS axis, both of which had to be tested). In the *P*2_1_2_1_2_1_ crystal, the two pairs of FAS molecules, which have the same orientation and which are related by a pure translation in the unit cell, generated strong peaks in the native Patterson map. The peaks were observed in two of the six Harker sections of space group *P*2_1_2_1_2_1_. They correspond to the vectors between centers of particles with the same orientation in the unit cell. The peak in the native Patterson map with fractional coordinates *u* = 0.46, *v* = −0.5, *w* = 0 and the formal Harker vector *u* = 2*x, v* = −0.5, *w* = 2*z* − 0.5 established the position of one FAS particle at *x* = 0.23, *z* = 0.25 with an arbitrary *y* component. The position along the *y* axis cannot be deduced from the native Patterson map because of the absence of strong cross-peaks between molecules with different orientations in the unit cell. Nonetheless, the translational search was reduced from a three-dimensional to a one-dimensional problem. We were unable to find correct translation-function solutions until we collected data sets that were complete at very low resolution (Fig. 3 ▶). Inclusion of essentially all low-angle reflections yielded outstanding peaks that were consistent in both space groups. Furthermore, the molecules translated according to this solution had reasonable packing in the unit cells.

Despite the molecular-replacement solutions, which we know *a posteriori* to be correct, phases calculated from the EM map could not successfully be extended to higher resolution. This was judged by anomalous difference Fourier map analysis with the aim of locating heavy atoms in data sets collected from derivatized crystals and visual inspection of electron-density maps for identification of α-­helical structures. Similar observations were reported for the phasing of yeast FAS crystals (Xiong, 2008 ▶), which may be explained by the limited resolution of the EM map and by the inaccuracy of low-resolution molecular-replacement solutions and consequently also the NCS operators for density averaging when applied to higher resolution data during phase extension. However, we also had to consider crystal twinning as one possible reason complicating the structure determination of *T. lanuginosus* FAS.

### 3.2. Phenomenology of imperfect pseudo-merohedral twinning

There were both theoretical considerations and experimental observations that suggested that the *P*2_1_ FAS crystal form was twinned. Twins are regular aggregates consisting of crystals of the same species joined together in some definite mutual orientation (Giacovazzo & Bolognesi, 1992 ▶). Given that the scattered waves from the individual crystalline domains do not interfere, which holds true if the domains are much larger than the coherence length of the X-ray beam, the resulting diffraction patterns can be described as a superposition of the diffraction pattern of each twin domain (Yeates, 1997 ▶; Parsons, 2003 ▶). The twin law defines the orientation of the different domains with respect to each other and the twin fraction determines their fractional contribution to the overall diffraction pattern (Herbst-Irmer & Sheldrick, 1998 ▶).

The theory of crystal twinning, termed geminography, has recently been reviewed (Grimmer & Nespolo, 2008 ▶) and comprehensive introductions to twinning in macromolecular crystallo­graphy are available (Yeates, 1997 ▶; Yeates & Fam, 1999 ▶; Parsons, 2003 ▶; Dauter, 2003 ▶). For crystals built from pure enantiomeric molecules, which is generally the case for biopolymers, merohedral twinning can occur if the point-group symmetry of the lattice (holohedry) contains one or more rotational symmetries that are not present in the symmetry of the diffraction pattern (Laue class). Possible merohedral twin laws can be derived by coset decomposition of the holohedry with respect to the Laue class (Flack, 1987 ▶). They give rise to an exact superposition of reciprocal lattices originating from two twin domains and consequently to a perfect overlap of all diffraction spots on the detector. Pseudo-merohedral twinning may occur when a crystal has a unit cell with such fortuitous geometry that its lattice emulates the holohedry of a crystal system with higher symmetry. In this case, rotational symmetry operations also exist as possible twin laws that are absent in the proper Laue class of the crystal but map the reciprocal-lattice points of twin domains onto each other. In the case of nonmerohedral twinning, the twin law is a rotational symmetry operation that does not belong to the holohedry or apparent holohedry of the crystal and therefore the reciprocal lattices of the twin domains do not overlap in all directions. Whereas nonmerohedral twinning introduces abnormalities into diffraction patterns, merohedral and pseudo-merohedral twinning are not visibly revealed in the diffraction pattern and may only be identified at the level of data scaling, statistical analysis of intensity distributions (Stanley, 1972 ▶; Britton, 1972 ▶; Murray-Rust, 1973 ▶; Rees, 1980 ▶; Yeates, 1997 ▶; Padilla & Yeates, 2003 ▶) and during subsequent steps of structure determination and model refinement (Redinbo & Yeates, 1993 ▶; Herbst-Irmer & Sheldrick, 1998 ▶; Yang *et al.*, 2000 ▶; Dauter, 2003 ▶).

**Figure 4:**
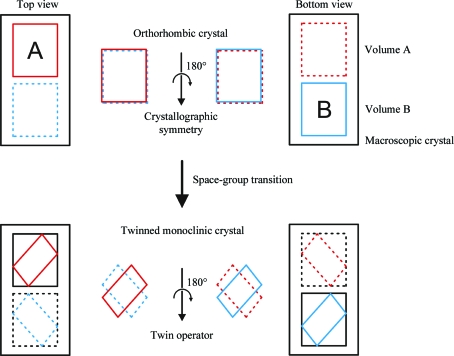
Crystal twinning through space-group transition from orthorhombic to monoclinic. A macroscopic crystal is shown from the bottom and top view. In the orthorhombic space group, the molecular packing within two volumes in the crystal, labeled A and B, is undistinguishable when viewed from top or bottom, because the orthorhombic crystallographic symmetry relates the content of the two volumes by a twofold (screw) axis. A twinned monoclinic crystal results if the molecules rearrange in the same manner in A as viewed from the top and in B as viewed from the bottom. The monoclinic packing is equivalent in the two volumes but is no longer related by crystallographic symmetry; instead, the twin operator, a 180° rotation around an axis in the plane perpendicular to the unique crystallographic *b* axis of the monoclinic lattice, relates the diffraction patterns of the two volumes.

Statistical analysis of the intensity distributions for data sets collected from *P*2_1_ crystals yielded inconclusive results regarding anomalies or the presence of possible twinning. Because the measured intensity distributions were compared with theoretical values expected from Wilson statistics, these tests may fall short with low-resolution data, for which Wilson scaling is not applicable. Aberrant results for intensity statistics can also be caused by anisotropic diffraction and pseudo-crystallographic symmetry (Padilla & Yeates, 2003 ▶). Furthermore, we observed significantly different intensity statistics after data processing using different software packages (data not shown). However, theoretical considerations prompted us to suspect crystal twinning, as illustrated in Fig. 4 ▶. When switching space group, the molecules rearrange and form at least some new crystal contacts. Unless this rearrangement occurs from a single nucleation point and then propagates in a concerted manner through the entire macroscopic crystal volume, transitions from orthorhombic to monoclinic space group inevitably yield a twinned crystal. With multiple nucleation points, the rearrangement from orthorhombic to monoclinic can occur in opposite directions that, because of orthorhombic symmetry, are energetically equivalent and therefore expected to occur with a statistical equal probability. The result is a twinned crystal that contains monoclinic domains that are related by a twin operator, as shown in Fig. 4 ▶.

**Figure 5:**
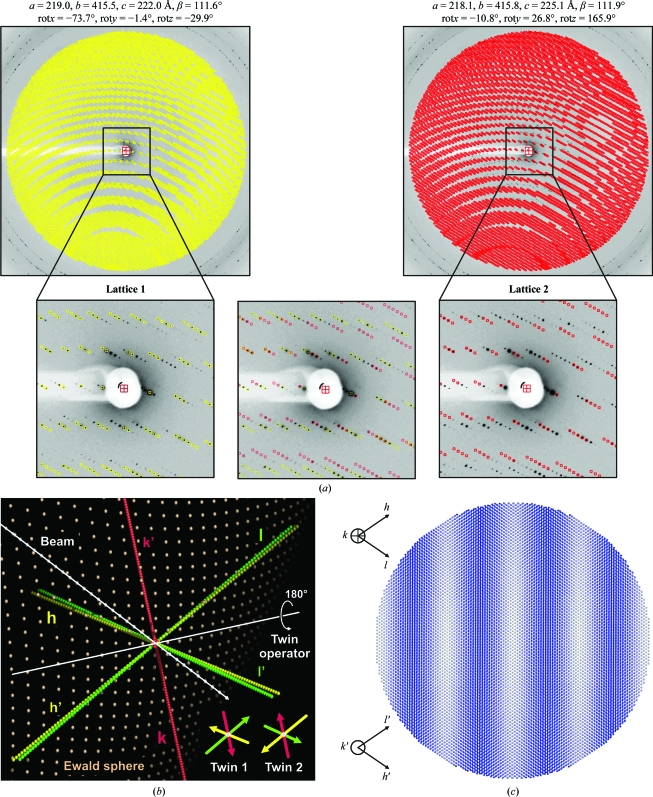
Monoclinic *P*2_1_ FAS crystals from *T. lanuginosus* are imperfectly pseudo-merohedrally twinned. (*a*) Example of a diffraction image with indexing of two monoclinic Bravais lattices. The unit-cell parameters and crystal orientation matrices were refined independently for both lattices. Predicted spots for the first lattice are shown in yellow, whereas predicted spots for the second lattice are shown in red. (*b*) Orientation of the two reciprocal twin lattices according to the crystal orientation matrices given in (*a*) with respect to the X-ray beam and the Ewald sphere. The two twins are related by a 180° rotation around an axis in the plane perpendicular to the monoclinic *b* axis. (*c*) Effect of twinning on spot overlap in the diffraction pattern. Reciprocal-lattice points for the section *h*0*l* (twin 1) and *l*′0*h*′ (twin 2) are shown to a resolution of 5 Å and colored using a gradient from white (maximum spot overlap) to blue (maximum spot separation).

When *T. lanuginosus* FAS crystals were dehydrated, we typically observed the appearance of cracks in the crystals under the microscope. This crystal cracking was reflected by abnormalities in the diffraction patterns. For some regions in reciprocal space the spots seemingly originated from a single crystal, but in other regions split spots were observed or even lattices that appeared to be from two crystals. Nevertheless, it was not obvious how to distinguish between simple crystal splitting or possible twinning until it became possible, for some images taken from *P*2_1_ crystals, to index two lattices in the same image and independently refine their parameters (Fig. 5 ▶ *a*). Both lattices showed the same underlying unit-cell parameters, but different rotation matrices, which define the crystal orientation in the beam. The relationship between the two lattices, namely twinning as illustrated in Fig. 4 ▶, was evident when we displayed them in three-dimensional space (Fig. 5 ▶ *b*). The twin operator, a 180° rotation around an axis in the plane perpendicular to the unique *b* axis, perfectly superimposes the b* axis onto itself and approximately aligns the a* axis of one twin domain with the c* axis of the other twin domain and *vice versa*.

Data from twinned crystals could be indexed with a *C*-­centered orthorhombic Bravais lattice, but in the real monoclinic unit cell the *a* axis is about 2% shorter than the *c* axis. Thus, there is no perfect superposition of the twin lattices in reciprocal space, as in the case of ‘exact’ pseudo-mero­hedral twinning (a term used by Dauter, 2003 ▶). Because here the monoclinic lattice only approximates higher *C*-centered orthorhombic symmetry, we refer to this type of twinning as ‘imperfect’ pseudo-merohedral twinning. The slightly different lengths of the *a* and *c* axes in the twinned FAS *P*2_1_ crystals generate an interference pattern of the two reciprocal lattices. Sections along *k* in reciprocal space, as shown for *h*0*l* in Fig. 5 ▶(*c*), are Moiré patterns containing alternating zones of almost perfectly overlapping, partially overlapping and well separated reflections.

### 3.3. Processing of data from twinned crystals

**Figure 6:**
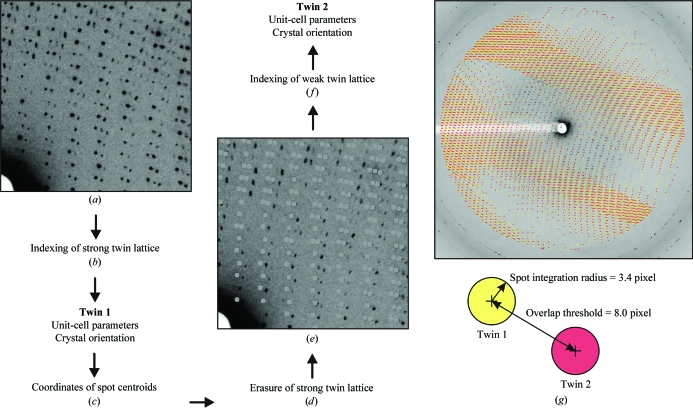
Processing of diffraction images collected from twinned crystals. (*a*) Section of a diffraction image of a twinned *P*2_1_ FAS crystal from *T. lanuginosus*. (*b*) For this particular crystal, the twin fraction was lower than 0.5 and therefore one of the two twin lattices was stronger and could be indexed. (*c*) Coordinates of all predicted spot centroids belonging to twin lattice 1 are obtained after refinement of the unit-cell parameters and crystal orientation. (*d*) All diffraction spots belonging to the strong twin lattice were erased in the raw image by replacing them with a gray circle with a value corresponding to the image background. (*e*) Diffraction image after spot erasure. (*f*) Refinement of the unit-cell parameters and crystal orientation of the weak twin lattice was then possible based on the remaining diffraction spots. (*g*) After integration of the two twin domains, overlapping and partially overlapping reflections were eliminated using the program *UNTANGLE* (Buts *et al.*, 2004 ▶). An overlap threshold was applied where reflections from the two twin domains were rejected if the distance between their spot centroids was closer than eight pixels. In the diffraction image shown, spatially separated reflections from the two twins are colored yellow and red, respectively. Alternating zones of overlapping and spatially separated reflections are visible.

The relative intensities of the two twin-related patterns varied among the different *P*2_1_ crystals tested. However, attempts to reduce the twinning by very slow crystal stabilization did not prove successful. We were also unable to collect data from single individual twin domains using highly focused X-ray beams with a microdiffractometer. To quantitatively assess the severity of the twinning, we followed the strategy outlined in Fig. 6 ▶. This allowed us to process data from both twin lattices in order to determine twin fractions and the percentage of the data that is actually affected by the twinning. Data from crystals with a twin fraction close to 0.5 could not be processed because the indexing routines of the data-reduction programs tended to oscillate between the two lattices during the processing of diffraction images. However, for crystals with a lower twin fraction we usually could index the stronger twin lattice, yielding its predicted spot centroids. To allow indexing of the weak twin lattice, all spots belonging to the strong lattice were erased in the raw diffraction images. This was accomplished by reading the coordinates of the strong lattice-spot centroids and replacing the corresponding spots in the images with a circle in the background gray value. Unit-cell parameters and crystal orientation could then be refined for the weak lattice based on the remaining spots. By keeping the previously determined parameters fixed, data were integrated for both twin domains from the original diffraction images. Overlapping and partially overlapping reflections were eliminated using the program *UNTANGLE* (Buts *et al.*, 2004 ▶). This yielded clean data for the two twin domains for subsequent scaling and merging (Table 1 ▶).

**Figure 7:**
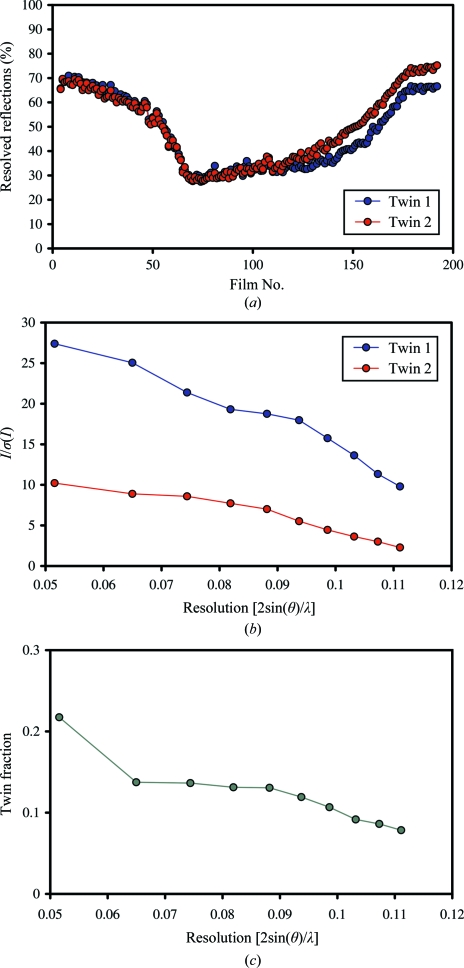
Analysis of data obtained from the two twins in the *P*2_1_ crystal form. (*a*) The percentage of reflections that are not affected by overlap or partial overlap depends on the orientation of the twinned crystal in the beam. Data were collected with a 0.5° oscillation range. (*b*) Resolution-dependence of the signal-to-noise ratio for the data sets of the two twins. (*c*) Resolution-dependence of the apparent twin fraction (TF), with TF = 〈*I*(*h*′)〉/[〈*I*(*h* *k* *l*)〉 + 〈*I*(*h*′*k*′*l*′)〉], where 〈*I*(*h*)〉 and 〈*I*(*h*′)〉 are the mean intensities for twin 1 and 2, respectively.

The percentage of data that were unaffected by spot overlap depended on the orientation of the crystal in the X-ray beam. Typically, 30–70% of spots per diffraction image were lost when the twins were untangled (Fig. 7 ▶ *a*), resulting in data sets with slightly higher then 50% completeness. The apparent twin fraction depended on the size and number of the twin domains in the beam and their relative diffraction limits. For the crystal whose twin analysis is presented here and which was used for the 5 Å resolution electron-density map of *T. lanuginosus* FAS (Jenni *et al.*, 2006 ▶), the relative contributions of the two twin domains to the overall diffraction were substantially different. Resolution-dependent intensity statistics for the clean data sets showed that the signal-to-noise ratio for the weaker twin approached 2 at 9 Å resolution, compared with about 10 for the stronger twin (Fig. 7 ▶ *b*). For this particular crystal, the apparent twin fraction calculated from the intensity statistics is greater than 0.2 at very low resolution but then drops to below 0.1 at 9 Å resolution (Fig. 7 ▶ *c*). The observed decrease in the apparent twin fraction at higher resolution suggests a difference in the average Wilson *B* factor for the two twin domains. We concluded from this analysis that twinning beyond 9 Å resolution is negligible, which justified using the complete data from the stronger twin without un­tangling in the final structure determination.

The described procedure yields clean data for accurate statistical analysis. However, a substantial amount of data is lost and completeness cannot be improved very much by scaling the data sets from both twins together (Table 1 ▶). We therefore screened for crystals with the lowest apparent twin fractions. Another possible strategy would be to fit two reflections that are closer than a certain cutoff in reciprocal space to a bimodal spot profile and to sum up their intensities during integration. This would artificially render partially overlapping reflections perfectly overlapping. This subset of intensities could then, in principle, be detwinned using the same algorithms as used for detwinning merohedrally and pseudo-meroherally twinned data (Yeates, 1997 ▶) with a twin fraction determined from the subset of non-overlapping reflections. However, the continuous spectrum in the separation of spot centroids makes data indexing and integration difficult (Bourgeois, 1999 ▶; Duisenberg *et al.*, 2003 ▶).

Considering the difficulties in extending the phases to higher resolution starting from the molecular-replacement solutions, we had to rely on heavy-atom phasing. Before we gained a detailed understanding of the nature of the twinning, we had collected several SAD data sets from derivatized *P*2_1_ crystals which proved unsuccessful in locating heavy-atom positions. We realised that the twinning would prevent us from accurately determining anomalous differences. Therefore, from understanding the system we devised a strategy that involved heavy-atom phasing in the poorly diffracting orthorhombic space group and phase extension using data from better diffracting but twinned crystals. The quantitative estimation of twin fractions for monoclinic crystals, as presented above, thereby guided us to include data for phase extension that were least affected by twinning.

## 4. Conclusions

Exact pseudo-merohedral twinning for crystals in space group *P*2_1_ with *a* and *c* unit-cell axes of equal length, where the symmetry of a *C*-centered orthorhombic Bravais lattice is emulated, has been reported for a number of macromolecular crystal structures (Ban *et al.*, 1999 ▶; Yang *et al.*, 2000 ▶; Wittmann & Rudolph, 2007 ▶; Sultana *et al.*, 2007 ▶; Heitmann & Einsle, 2008 ▶). Recent surveys of the PDB indicated additional candidates that are potentially affected by this type of twinning (Lebedev *et al.*, 2006 ▶; Wittmann & Rudolph, 2007 ▶). More widely applied methods to improve crystal diffraction by post-crystallization treatments (Heras & Martin, 2005 ▶) may lead to similar cases of imperfect pseudo-merohedral twinning in the future, as described here for the space-group transition from an orthorhombic crystal with pseudo-*C*-centered sym­metry to a monoclinic crystal. Further developments in crystallo­graphic software should allow us to better deal with this type of twinning during data integration and provide practical methods for detwinning and structure refinement. We were unable to draw convincing conclusions from intensity distribution analysis and twinning tests. The *P*2_1_ crystals presented here possess rotational pseudo-symmetry, which could lead to difficulties in detecting twinning (Lebedev *et al.*, 2006 ▶). Our observations corroborate the importance, if there are problems with structure determination, of visually in­specting the diffraction images carefully and considering the substantial information that molecular-replacement solutions, self-rotation function analysis and heavy-atom positions can reveal about the possible occurrence and nature of twinning.

## Figures and Tables

**Table 1:** Data-processing statistics for the twinned *P*2_1_ *T. lanuginosus* FAS crystal Values in parentheses are for the highest resolution shell.

	Twin 1	Twin 2	Merged
Unit-cell parameters (Å, °)	*a* = 217, *b* = 414, *c* = 222, β = 111.6	*a* = 215, *b* = 416, *c* = 222, β = 112.0	*a* = 217, *b* = 414, *c* = 222, β = 111.6
Space group	*P*2_1_	*P*2_1_	*P*2_1_
Wavelength (Å)	1.0000	1.0000	1.0000
Resolution (Å)	100.0–9.0 (9.3–9.0)	100.0–9.0 (9.3–9.0)	100.0–9.0 (9.3–9.0)
Unique reflections	10067	9665	14037
Redundancy	2.3	2.1	3.1
*I*/σ(*I*)	18.8 (9.8)	6.9 (2.3)	3.9 (1.5)
*R*_linear_† (%)	3.8 (6.0)	11.4 (41.9)	11.0 (19.8)
Completeness (%)	36.7 (28.4)	35.1 (26.9)	51.0 (45.8)

† *R*_linear_ = 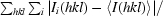 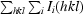, where 〈*I*(*hkl*)〉 is the mean intensity of the reflections.

## References

[bb1] Ban, N., Nissen, P., Hansen, J., Capel, M., Moore, P. B. & Steitz, T. A. (1999). *Nature (London)*, **400**, 841–847. 10.1038/23641 10476961

[bb2] Bourgeois, D. (1999).* Acta Cryst.* D**55**, 1733–1741. 10.1107/s0907444999008355 10531523

[bb3] Britton, D. (1972).* Acta Cryst.* A**28**, 296–297.

[bb4] Buts, L., Dao-Thi, M.-H., Wyns, L. & Loris, R. (2004). *Acta Cryst.* D**60**, 983–984. 10.1107/S0907444904007048 15103159

[bb5] Dauter, Z. (2003). *Acta Cryst.* D**59**, 2004–2016. 10.1107/s0907444903021085 14573956

[bb6] Duisenberg, A. J. M., Kroon-Batenburg, L. M. J. & Schreurs, A. M. M. (2003). *J. Appl. Cryst.* **36**, 220–229.

[bb7] Flack, H. D. (1987). *Acta Cryst.* A**43**, 564–568.

[bb8] Giacovazzo, C. & Bolognesi, M. (1992). *Fundamentals of Crystallography.* Oxford/Chester: Oxford University Press/International Union of Crystallography.

[bb9] Grimmer, H. & Nespolo, M. (2008). *Z. Kristallogr.* **221**, 28–50.

[bb10] Hackenjos, W. A. & Schramm, H. J. (1987). *Biol. Chem. Hoppe-Seyler*, **368**, 19–36. 10.1515/bchm3.1987.368.1.19 3548748

[bb11] Heitmann, D. & Einsle, O. (2008). *Acta Cryst.* D**64**, 993–999. 10.1107/S0907444908019197 18703849

[bb12] Heras, B. & Martin, J. L. (2005). *Acta Cryst.* D**61**, 1173–1180. 10.1107/S0907444905019451 16131749

[bb13] Herbst-Irmer, R. & Sheldrick, G. M. (1998). *Acta Cryst.* B**54**, 443–449.

[bb14] Hirshfeld, F. L. (1968). *Acta Cryst.* A**24**, 301–311.

[bb15] Jenni, S., Leibundgut, M., Boehringer, D., Frick, C., Mikolasek, B. & Ban, N. (2007).* Science*, **316**, 254–261. 10.1126/science.1138248 17431175

[bb16] Jenni, S., Leibundgut, M., Maier, T. & Ban, N. (2006). *Science*, **311**, 1263–1267. 10.1126/science.1123251 16513976

[bb17] Johansson, P., Wiltschi, B., Kumari, P., Kessler, B., Vonrhein, C., Vonck, J., Oesterhelt, D. & Grininger, M. (2008). *Proc. Natl Acad. Sci. USA*, **105**, 12803–12808. 10.1073/pnas.0805827105 PMC2529065 18725634

[bb18] Kabsch, W. (1993). *J. Appl. Cryst.* **26**, 795–800.

[bb19] Kolodziej, S. J., Penczek, P. A. & Stoops, J. K. (1997). *J. Struct. Biol.* **120**, 158–167. 10.1006/jsbi.1997.3911 9417980

[bb20] Lebedev, A. A., Vagin, A. A. & Murshudov, G. N. (2006). *Acta Cryst.* D**62**, 83–95. 10.1107/S0907444905036759 16369097

[bb21] Leibundgut, M., Jenni, S., Frick, C. & Ban, N. (2007). *Science*, **316**, 288–290. 10.1126/science.1138249 17431182

[bb22] Lomakin, I. B., Xiong, Y. & Steitz, T. A. (2007). *Cell*, **129**, 319–332. 10.1016/j.cell.2007.03.013 17448991

[bb23] Maier, T., Jenni, S. & Ban, N. (2006). *Science*, **311**, 1258–1262. 10.1126/science.1123248 16513975

[bb24] Maier, T., Leibundgut, M. & Ban, N. (2008). *Science*, **321**, 1315–1322. 10.1126/science.1161269 18772430

[bb25] Matthews, B. W. (1968). *J. Mol. Biol.* **33**, 491–497. 10.1016/0022-2836(68)90205-2 5700707

[bb26] Mueller, M., Jenni, S. & Ban, N. (2007). *Curr. Opin. Struct. Biol.* **17**, 572–579. 10.1016/j.sbi.2007.09.004 17964135

[bb27] Murray-Rust, P. (1973). *Acta Cryst.* B**29**, 2559–2566.

[bb28] Oesterhelt, D., Bauer, H. & Lynen, F. (1969). *Proc. Natl Acad. Sci. USA*, **63**, 1377–1382. 10.1073/pnas.63.4.1377 PMC223475 5260941

[bb29] Otwinowski, Z. & Minor, W. (1997).* Methods Enzymol.* **276**, 307–326. 10.1016/S0076-6879(97)76066-X 27754618

[bb30] Padilla, J. E. & Yeates, T. O. (2003).* Acta Cryst.* D**59**, 1124–1130. 10.1107/s0907444903007947 12832754

[bb31] Parsons, S. (2003). *Acta Cryst.* D**59**, 1995–2003. 10.1107/s0907444903017657 14573955

[bb32] Redinbo, M. R. & Yeates, T. O. (1993). *Acta Cryst.* D**49**, 375–380. 10.1107/S090744499300294X 15299512

[bb33] Rees, D. C. (1980). *Acta Cryst.* A**36**, 578–581.

[bb34] Stanley, E. (1972). *J. Appl. Cryst.* **5**, 191–194.

[bb35] Sultana, A., Alexeev, I., Kursula, I., Mäntsälä, P., Niemi, J. & Schneider, G. (2007). *Acta Cryst.* D**63**, 149–159. 10.1107/S0907444906044271 17242508

[bb36] Tong, L. & Rossmann, M. G. (1990). *Acta Cryst.* A**46**, 783–792. 10.1107/s0108767390005530 2174243

[bb37] Tong, L. & Rossmann, M. G. (1997). *Methods Enzymol.* **276**, 594–611. 9048382

[bb38] Wittmann, J. G. & Rudolph, M. G. (2007). *Acta Cryst.* D**63**, 744–749. 10.1107/S0907444907016605 17505114

[bb39] Xiong, Y. (2008). *Acta Cryst.* D**64**, 76–82. 10.1107/S090744490705398X PMC2394795 18094470

[bb40] Yang, F., Dauter, Z. & Wlodawer, A. (2000). *Acta Cryst.* D**56**, 959–964. 10.1107/s0907444900007162 10944332

[bb41] Yeates, T. O. (1997). *Methods Enzymol.* **276**, 344–358. 9048378

[bb42] Yeates, T. O. & Fam, B. C. (1999). *Structure*, **7**, R25–R29. 10.1016/S0969-2126(99)80016-1 10368291

